# Pulmonary fibrosis in dyskeratosis congenita: a case report with a PRISMA-compliant systematic review

**DOI:** 10.1186/s12890-021-01645-w

**Published:** 2021-09-03

**Authors:** Ping Wang, Zuojun Xu

**Affiliations:** Department of Respiratory and Critical Care Medicine, Peking Union Medical College Hospital, Chinese Academy of Medical Sciences and Peking Union Medical College, Beijing, China

**Keywords:** Dyskeratosis congenita, Pulmonary fibrosis, Clinical features, Gene mutations, Prognosis, Systematic review, Case report

## Abstract

**Background:**

Dyskeratosis congenita (DC) is a rare genetic disorder of poor telomere maintenance. Pulmonary fibrosis (PF) related to DC is rarely reported.

**Case presentation:**

A 23-year-old student presented with a four-year history of progressive cough and exertional dyspnea. Physical examination was remarkable for typical mucocutaneous abnormalities. Chest computerized tomography scan revealed interstitial fibrosis. Testing of peripheral blood leukocytes confirmed that his telomeres were 30th percentile of age-matched controls. A heterozygous missense mutation located in exon 22 of PARN gene was identified in the patient by whole exome sequencing. The patient refused danazol therapy and lung transplantation, and died of respiratory failure 2 years later. In addition, this case and 26 reported cases of DC-related PF identified through the comprehensive search of PubMed, Web of Science, WANFANG and CNKI were reviewed. Later-onset PF was observed in 11 patients (40.7%). Radiological usual interstitial pneumonia (UIP) pattern or possible UIP pattern was noted only in half of patients. However, histopathological UIP or probable UIP patterns were found in 63.6% of patients. Age at bone marrow failure (BMF) and the frequency of normal to mild thrombocytopenia in later-onset patients was significantly higher than in early-onset patients (p = 0.017 and p = 0.021, respectively). Age at PF and age at BMF in DC patients with TERC/TERT variants was significantly higher than in those with TINF2 variants or DKC1/NHP2 variants (p = 0.004 and p = 0.003, respectively). The patients with TERT/TERC/RTEL1/PARN variants had a significantly better transplant-free survival than those with TINF2 variants or DKC1/NHP2 variants (p < 0.05). Patients who underwent surgical lung biopsy had significantly worse transplant-free survival than those without lung biopsy (p = 0.042). Worse survival was found in patients with immunosuppression therapy than in those without (p = 0.012).

**Conclusions:**

It is common for DC-associated PF to occur later in life without significant hematological manifestations. Mutations in the genes encoding different components of the telomere maintenance pathway were associated with clinical phenotypes and prognosis. PF caused by DC should be kept in mind by clinicians in the differential diagnosis of patients with unexplained PF and should be excluded before diagnostic surgical lung biopsy is undertaken or empirical immunosuppression therapy is prescribed.

**Supplementary Information:**

The online version contains supplementary material available at 10.1186/s12890-021-01645-w.

## Background

Short telomere syndromes are multisystem disorders with widespread clinical manifestations associated with shortened telomere length. Dyskeratosis congenita (DC) is the archetypal short telomere syndromes cause by germline mutations in telomere maintenance genes (TMG), which is characterized by the presence of the mucocutaneous triad of abnormal skin pigmentation, nail dystrophy, and leucoplakia, associated with bone marrow failure (BMF) [1]. A wide spectrum of somatic features affecting every system in the body, including pulmonary fibrosis (PF), liver cirrhosis, and premature hair graying, have been associated with DC. About 20% of DC patients develop PF [2]. The abnormal mucocutaneous changes usually appear first, often before the age of 10 years, and BMF develops frequently before the age of 20 [3]. PF usually develops after the appearance of skin abnormalities and BMF. However, the appearances, age at onset, and disease severity of these features are highly variable [4]. The interstitial lung abnormalities seen on chest computed tomography (CT) often show a usual interstitial pneumonia (UIP) pattern, and histological UIP pattern has accordingly been described in previously reported cases. However, atypical radiological and histopathological features for UIP have also been reported [5]. Telomere maintenance relies on a complex interaction between the telomerase complex (telomere elongation), the shelterin complex (telomere end protection), and the CST complex (telomere capping). With the great progression of gene sequencing techniques in the last decade, about 60% of DC patients are now classified as having mutations in known genes in the telomere maintenance pathway, including those impacting telomerase enzyme activity (TERC, TERT), telomerase RNA mutation (PARN), DNA helicase activity (RTEL1), telomerase cofactors (DKC1, NHP2, NOP10, NAF1, GAR1, TCAB1), shelterin function (TINF2, ACD), and the CST complex (CTC1). It is not known how these gene mutations correlate with clinical phenotypes of DC-related PF patients. There is no standard treatment for PF in DC. Danazol, immunosuppression (IS) therapy, and lung transplantation have been reported as being used in DC-related PF patients. Patients with clinically symptomatic PF had a rapidly progressive disease with a median survival time less than 3 years after diagnosis [6,7]. DC-related PF accounts for 15–20% of fatalities in DC [7]. Here we reported a rare case of DC-related PF and reviewed all published cases in the literature to review the clinical, genotypic characteristics and prognosis of DC-related PF. Addressing these questions may help clinicians improve the understanding and management of PF associated with DC.

## Case presentation

### Case

**Fig. 1:**
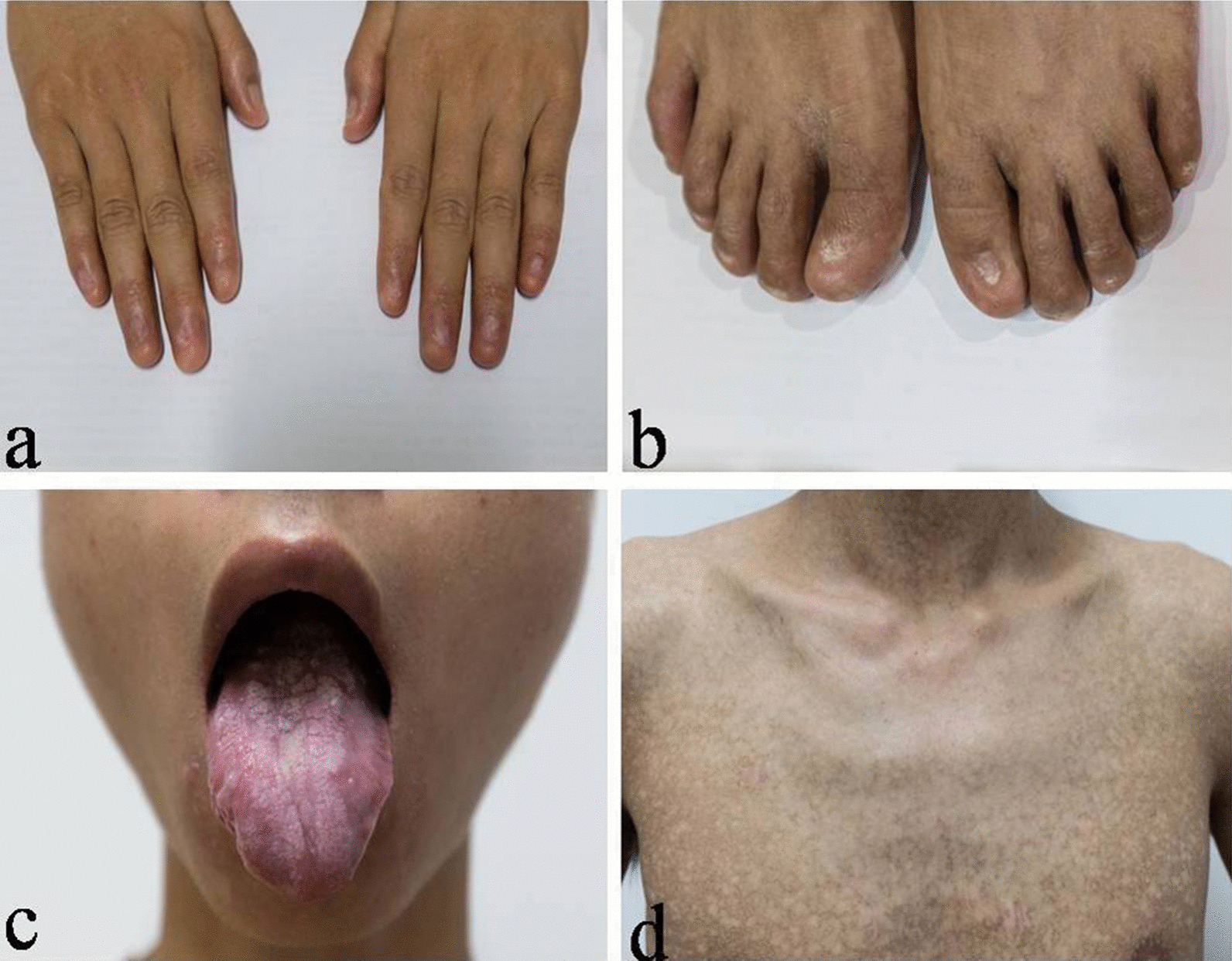
Typical mucocutaneous changes in a 23-year-old man: **a**, **b** Atrophic and dystrophic finger and toe nails. **c** Leukoplakia on the tongue. **d** Fine reticular pigmentation on the body

**Fig. 2:**
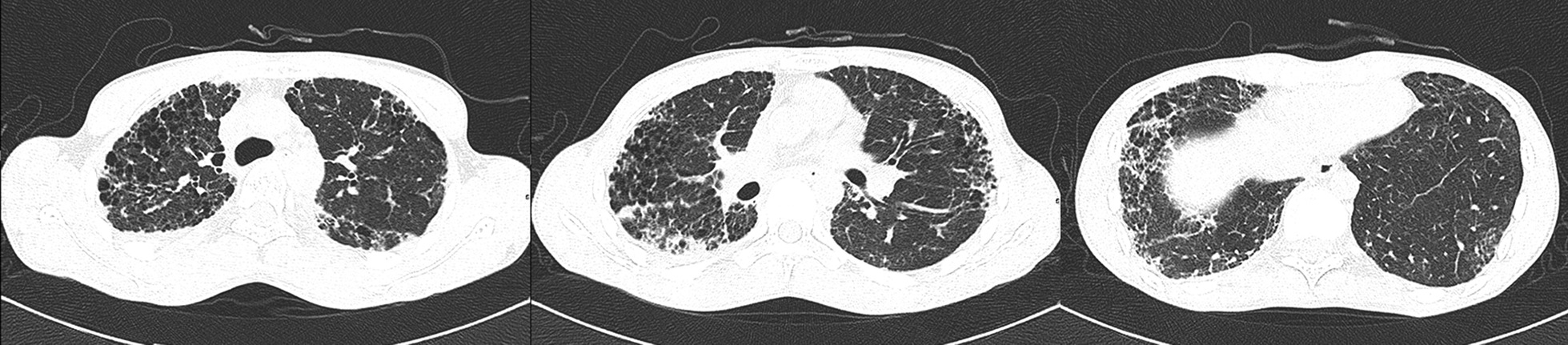
High resolution computed tomography manifestation of the patient. Chest high resolution computed tomography scan showed mid- and upper-zone predominant bilateral peripheral reticulolinear shadows, interlobular septal thickening with associated traction bronchiectasis and honeycombing, compatible with pulmonary fibrosis

A 23-year-old student presented with a 4-year history of progressive cough and dyspnea on exertion. He was a non-smoker. Skin and nail changes and epiphora were noticed when he was 6 years old, but no diagnosis was made. His family history was unremarkable. Physical examination was remarkable for nail atrophy (Fig. 1a, b), tongue leukoplakia (Fig. 1c), and reticular skin pigmentation across his body (Fig. 1d). Chest CT scans revealed mid- and upper-zone–predominant subpleural interstitial changes consistent with interstitial fibrosis (Fig. 2). The rheumatology-associated antibody titers were negative, including the antinuclear, anti-dsDNA, anti-neutrophil cytoplasmic, and anti-extractable nuclear antigen antibodies. The complete blood count showed mild thrombocytopenia (platelets 86 ~ 101 × 10^9^/l). The examination of bone marrow smear and biopsy was normal. His arterial blood gas results were normal, but pulmonary function tests revealed a forced vital capacity of 1.84 L (38%), forced expiratory volume in 1 s of 1.82 L (44%), total lung capacity of 3.28 L (51%), forced expiratory volume in1 sec/forced vital capacity of 99%, and a markedly decreased diffusing capacity for carbon monoxide at 47% of predicated. He was diagnosed with DC based on the classic mucocutaneous triad and the presence of hematological abnormalities, PF, and epiphora. Quantitative polymerase chain reaction analysis according to the protocol [8] revealed a telomere length reduction in peripheral blood mononuclear cells at the 30th percentile of age-matched controls. A heterozygous mutation (c.1603 G>A) located in exon 22 of PARN gene (NM_001242992) that changed glycine to arginine (Gly535Arg) was identified in the patient by whole exome sequencing and was verified with Sanger sequencing. The patient refused danazol therapy and lung transplantation, and died of respiratory failure 2 years later.

### Literature search strategy and results

**Fig. 3:**
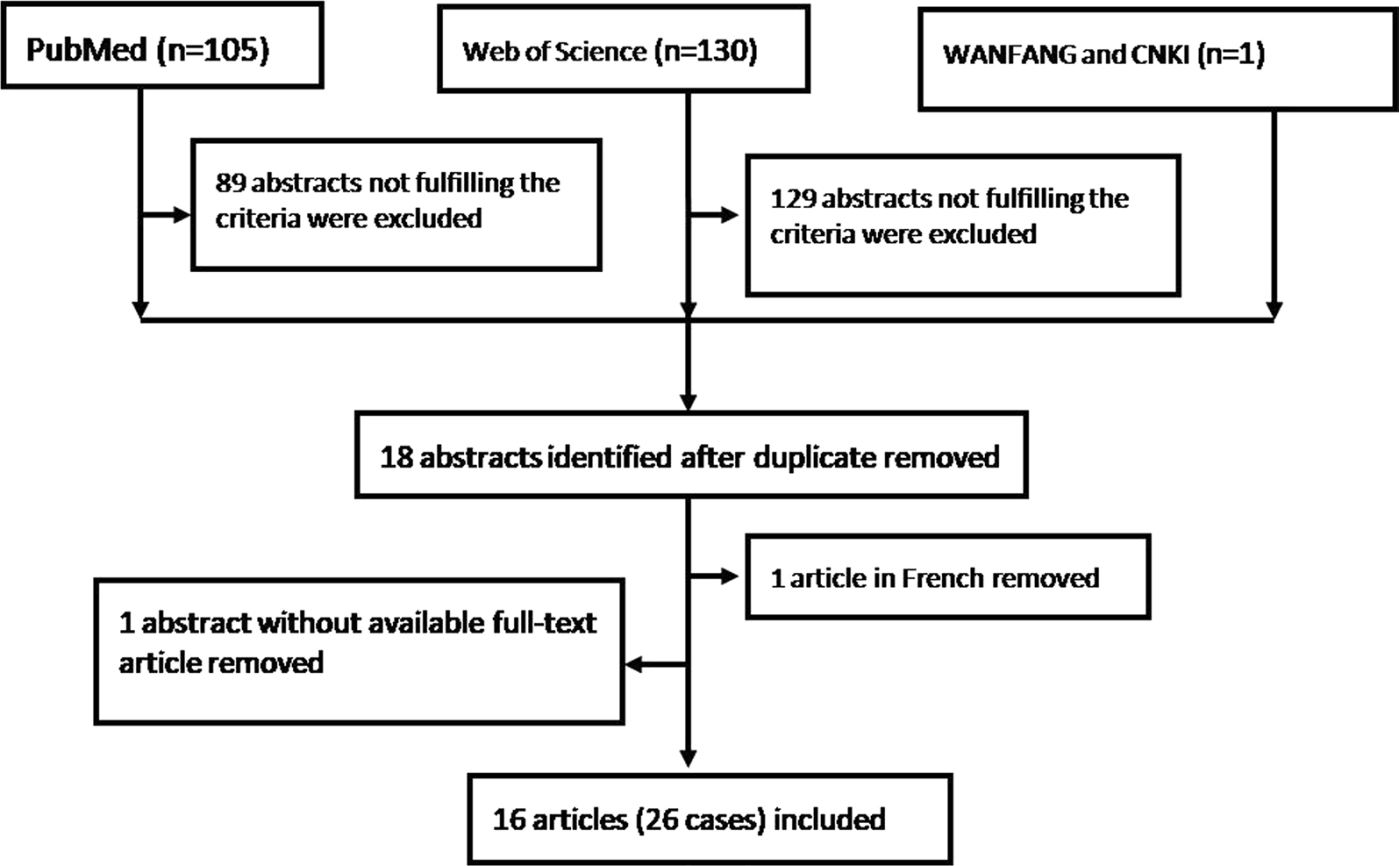
Procedure of publication retrieval and inclusion and exclusion of cases displayed in a flow chart

A search of PubMed, Web of Science, WANFANG, and China National Knowledge Infrastructure (CNKI) using the key words “dyskeratosis congenita” AND “pulmonary fibrosis” in the title and abstract was conducted on March 2020. No language limit or time span was set for the searches, which yielded 105 titles from PubMed, 130 titles from Web of Science, one title from WANFANG, and none from CNKI. Titles and abstracts were reviewed independently by two authors. All disagreements were resolved through discussion between the two reviewers. Only case reports or case series of DC patients with PF were included. Clinical diagnoses of DC were based on 1) the presence of at least two of the four major features (abnormal skin pigmentation, nail dystrophy, leukoplakia, and BMF) or one major feature plus a family history of DC and identification of germline pathogenic variants in known DC-related genes and 2) the presence of two or more of the other somatic features known to occur in DC, including pulmonary disease, epiphora, developmental delay, extensive dental caries/loss, premature hair loss/graying/sparse eyelashes, and liver disease [9]. PF was diagnosed by characteristic interstitial lung findings on chest CT. Consequently, 89 abstracts from PubMed and 129 abstracts from Web of Science that did not fulfill the inclusion criteria were excluded, and18 qualifying abstracts were identified. One abstract without available full text for data extraction and one article not in English or Chinese were removed, leaving16 full-text articles on a total of 26 patients to be included. Figure 3 illustrates the procedure of publication retrieval and the inclusion and exclusion of cases in a flow chart. The following data was extracted from eligible cases and recorded on a standard data extraction form: age at PF was defined as the age at the presentation of respiratory symptoms (chronic cough and/or exertional dyspnea); based on the age at PF, the patients were classified into early-onset (less than 40 years old at PF) and later-onset (more than 40 years old at PF); age at BMF was defined as the age at the initial diagnosis of BMF; BMF was categorized as mild (mild thrombocytopenia, untreated), moderate (multilineage cytopenia, untreated or treated with medication), and severe (pancytopenia requiring blood transfusion or hematopoietic stem cell transplantation); sex, history of tobacco use, family history, and symptoms; radiological and histopathological findings, pulmonary function test results; TMG mutations; treatment and outcome (death or lung transplantation). The findings of the chest CT images were classified as a UIP pattern (bilateral peripheral and basal-predominant reticulation and honeycombing, with or without traction bronchiectasis), possible UIP pattern (bilateral peripheral and basal-predominant reticulation and no findings inconsistent with UIP), pattern inconsistent with UIP (bilateral mid- and/or upper-zone–predominant subpleural reticulations or the presence of cysts), or compatible with nonspecific interstitial pneumonia (NSIP) pattern (bilateral diffuse reticulation and ground grass opacities). The histopathological findings were classified as UIP pattern, probable UIP pattern, possible UIP pattern, or not-UIP pattern [10].

All data analyses were conducted using IBM SPSS Statistics Version 19 (IBM Corp., Armonk, NY, USA). Continuous data was compared using independent samples *t* tests or ANOVAs as appropriate. Categorical variables were compared using chi-squared tests. P-values less than 0.05 were considered statistically significant. Kaplan–Meier survival curves were constructed, and the significance of survival stratification was tested using the log-rank test. Time to death or lung transplantation was calculated from the diagnosis date of DC-related PF.

Including our case, we identified a total of 27 patients with DC-related pulmonary PF [5–7,11–23]. Baseline demographics and clinical characteristics are shown in Table 1. The median time from BMF to PF was 13 (range: 6–26) years. Of the 24 patients with available data from hematological tests, nine (37.5%) showed normal complete blood count to mild thrombocytopenia.

**Table. 1:** Demographics and clinical characteristics of patients with DC-related PF (n = 27)

	Data
Age at PF (years)	32 (8–65)
Age at BMF (years)	20 (1–65)
Male	13/18 (72.2%)
Smoker	8/17 (47.1%)
BMF	20/24(83.3%)
Mild	5 (25%)
Moderate	3 (15%)
Severe	12 (60%)
DC triad features*	
0–1	3/18(16.7%)
2–3	15/18 (83.3%)
Family history of DC	8/16 (50%)
PFT	
FEV1, %pred ^†^	47 ± 15
FVC, %pred ^‡^	47 ± 20
FEV1/FVC ratio^§^	93 ± 7
TLC, %pred^§^	54 ± 14
DLCO, %pred^||^	38 ± 14

Data are presented as mean (range) or mean ± standard deviation or number/total number of patients with available data (%). PF: pulmonary fibrosis; BMF: bone marrow failure

DC: dyskeratosis congenital; PFT: pulmonary function test; FEV1: forced expiratory volume in 1 s; FVC: forced vital capacity; TLC: total lung capacity; DLCO: diffusing capacity for carbon monoxide

*DC triad features refer to oral leukoplakia, dysplastic nails and abnormal skin pigmentation

^†^FEV1 was available in 9 patients

^‡^FVC was available in 11 patients

^§^FEV1/FVC and TLC was available in 8 patients

^||^DLCO was available in 15 patients

**Fig. 4:**
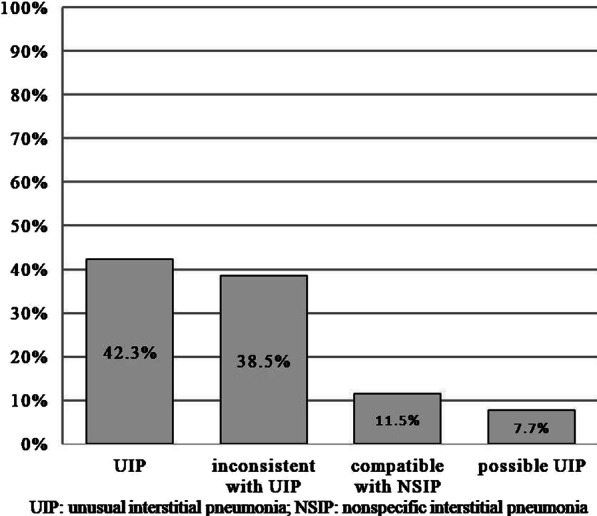
Frequency of radiological patterns on chest CT of patients with DC-related PF (n = 26). The bars indicated the frequencies of each radiological pattern with the percentage displayed within the bars

Of these 27 patients, 26 patients were classified into UIP, possible UIP, inconsistent with UIP and compatible with NSIP patterns on chest CT (Fig. 4). Details of chest CT manifestations in DC-related PF were described in 25 cases. The bilateral reticulation predominantly involved the lower lungs in the craniocaudal dimension in 12 cases (48.0%), the upper and/or middle lungs in 7 cases (28.0%), and the diffuse lungs in 6 cases (24.0%). Honeycombing was reported in 11 cases (44.0%), traction bronchiectasis in 12 cases (48.0%), and cysts in 5 cases (20.0%). No peribronchovascular predominance, profuse micronodules, or diffuse mosaic attenuation was found.

Of the12 patients who underwent surgical lung biopsy or autopsy, detailed histopathological descriptions were available for 11. UIP was found in 6 patients (54.5%), not-UIP in 3 (27.3%), probable UIP in 1 (9.1%), and possible UIP in 1 (9.1%). 3 patients with histological not-UIP pattern revealed prominent lymphatic proliferation in the fibrotic interlobular septa and in the bronchovascular sheaths, extensive bronchiolitis obliterans, or a mixed inflammatory cell infiltrate occupied the bronchioles and alveoli with some bronchioles obliterated with fibrinous exudates in the background of interstitial fibrosis. The relationships between CT patterns and histopathological patterns are shown in Table 2. Of the 6 cases inconsistent with UIP or compatible with NSIP on chest CT, 4 cases revealed histopathological UIP, while 2 cases revealed not-UIP based on the presence of marked lymphatic proliferation or bronchiolitis obliterans.

**Table. 2:** Relationship between radiological patterns and histopathological patterns (n = 10)

CT patterns	Histopathological patterns			
	UIP (n = 6)	Probable UIP (n = 1)	Not-UIP (n = 2)	Possible UIP (n = 1)
UIP (n = 3)	3	0	0	0
Possible UIP (n = 1)	0	0	0	1
Compatible with NSIP (n = 2)	0	1	1	0
Inconsistent with UIP (n = 4)	3	0	1	0

UIP: unusual interstitial pneumonia; NSIP: nonspecific interstitial pneumonia;

Later-onset PF was observed in 11 patients (40.7%). Age at BMF and the frequency of normal to mild thrombocytopenia in later-onset patients was significantly higher than in early-onset patients (p = 0.017 and p = 0.021, respectively) (Table 3). The details of TMG mutations were available in 19 cases. TINF2 was found in 6 cases (31.6%), TERC and/or TERT (TERC/TERT) in 5 cases (26.3%), DKC1 in 4 cases (21.1%), PARN in 2 cases (10.5%), RTEL1 in 1 case (5.3%), and NHP2 in 1 case (5.3%). Age at PF in DC patients with TERC/TERT variants was significantly higher than in those with TINF2 variants or those with DKC1 or NHP2 (DKC1/NHP2) variants (p = 0.004; Table 3). No differences in radiological patterns were found between early-onset PF patients and later-onset PF patients, or among patients with TERC/TERT variants, TINF2 variants and DKC1/NHP2 variants (*P* > 0.05; Table 3).

**Table. 3:** Clinical characteristics of patients with early-onset or later-onset PF (n = 27) and with different gene mutations (n = 16)

Variable	Early-onset (n = 16)	Later-onset (n = 11)	*P*	TERC/TERT (n = 5)	TINF2 (n = 6)	DKC1/NHP2 (n = 5)	*P*
Age at BMF* (year)	9 ± 8	30 ± 23	***0.017***	43 ± 14	4 ± 4	23 ± 21	***0.003***^†^
Age at PF (year)	/	/		56 ± 8	24 ± 13	36 ± 16	***0.004***^‡^
BMF (moderate to severe)	13/16 (81.3)	2/8 (25.0)	***0.021***	1/2 (50.0)	4/6 (66.7)	3/5 (60.0)	1.000
CT patterns							
UIP/possible UIP	7/15 (46.6)	6/11 (54.5)	1.000	4/5 (80.0)	3/6 (50.0)	2/5 (40.0)	> 0.05^#^

Data are presented as mean ± standard deviation or number/total number of the patients with available data (%)

DC: dyskeratosis congenital; BMF: bone marrow failure; PF: pulmonary fibrosis; CT: computed tomography; UIP: unusual interstitial pneumonia;/: not applicable

*Age at BMF was unavailable in 1 patient with later-onset PF and 1 patient with TINF2 variants

^†^TERC/TERT versus TINF2, *P* = 0.006; TINF2 versus DKC1/NHP2, *P* = 0.277; TERC/TERT versus DKC1/NHP2, *P* = 0.266

^‡^TERC/TERT versus TINF2, *P* = 0.001; TINF2 versus DKC1/NHP2, *P* = 0.137; TERC/TERT versus DKC1/NHP2, *P* = 0.028

^#^TERC/TERT versus TINF2, *P* = 0.545; TINF2 versus DKC1/NHP2, *P* = 1.000; TERC/TERT versus DKC1/NHP2, *P* = 0.524

**Fig. 5:**
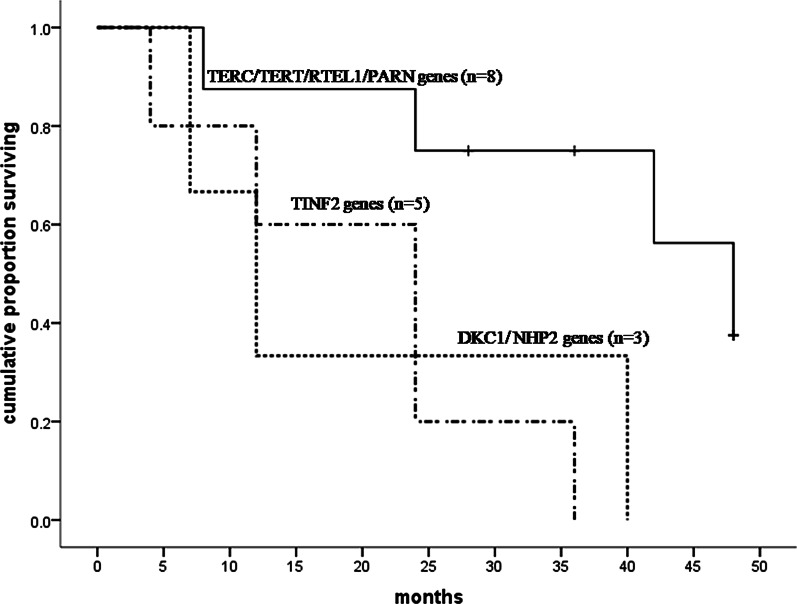
Kaplan–Meier survival analysis of telomere maintenance gene mutations in DC-related PF patients. Patients with mutations in TINF2 gene or DKC1/NHP2 gene had significantly worse transplant-free survival than those with variants in TERC/TERT/RTEL1/PARN gene (TINF2 gene vs TERC/TERT/RTEL1/PARN gene p = 0.013; DKC1/NHP2 gene vs TERC/TERT/RTEL1/PARN gene p = 0.027); No survival difference between TINF2 gene and DKC1/NHP2 gene was found (p = 0.684)

**Fig. 6:**
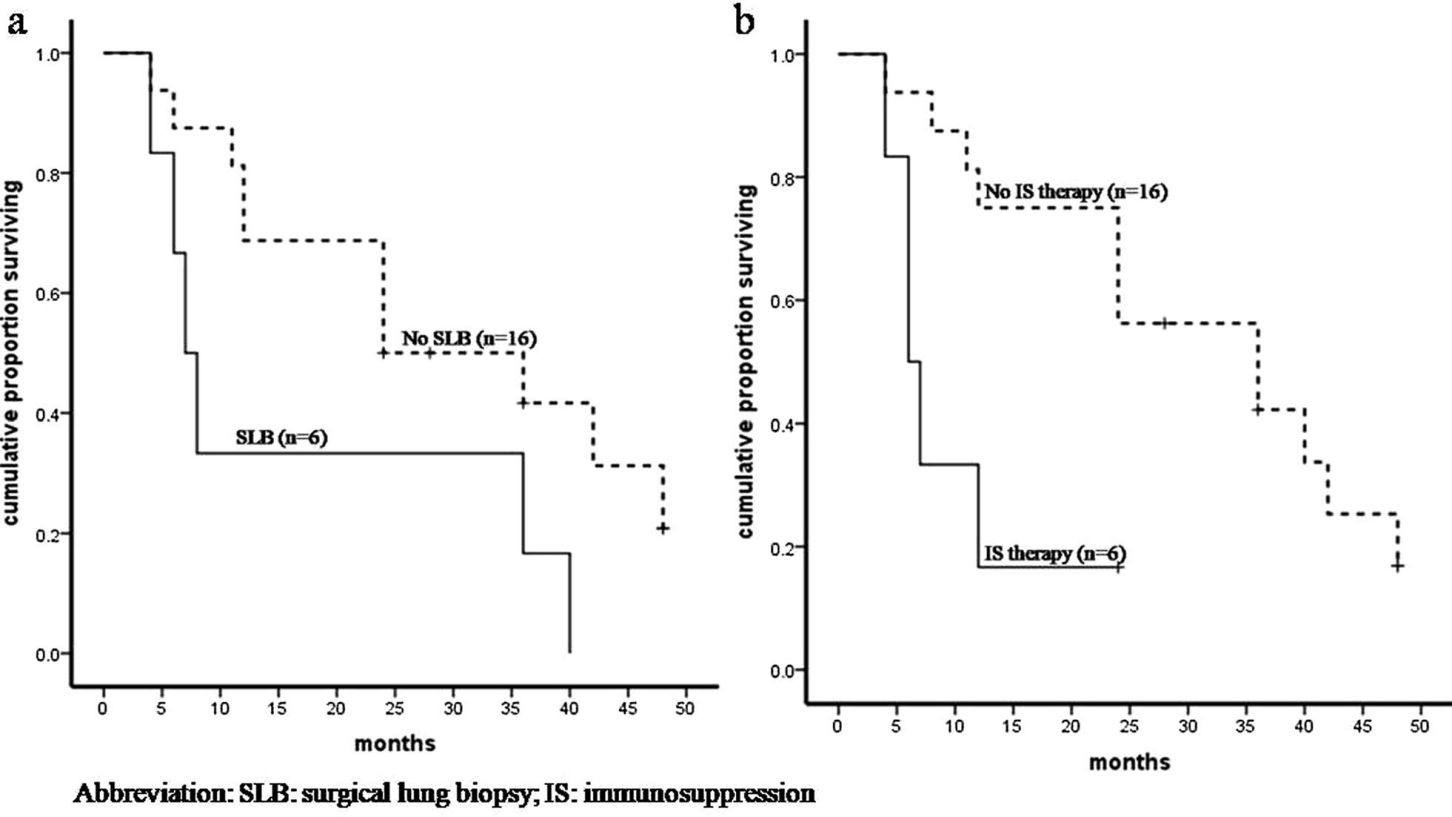
Kaplan–Meier survival analysis of surgical lung biopsy and immunosuppression therapy in DC-related PF patients: **a** patients who underwent surgical lung biopsy (“SLB”) had significantly worse transplant-free survival than those without (p = 0.042). **b** A worse survival was found in the patients with immunosuppression (“IS”) therapy compared to those without (p = 0.012)

The mean post-diagnosis follow-up period of the 22 patients with available follow-up data was 24 months (range: 4–48 months). There were 16 deaths and 1 lung transplantation. There were no differences in radiological patterns, pulmonary function measurements, severity of BMF, IS therapy or surgical lung biopsy (SLB) among patients with mutations in the TERC, TERT, RTEL1, or PARN (TERC/TERT/RTEL1/PARN) gene, TINF2 gene and DKC1/NHP2 gene (Additional file 1: Table S1). The median transplantation-free survival time was 24 months for the whole cohort; 48 months for patients with mutations in the TERC/TERT/RTEL1/PARN gene; 24 months with mutations in the TINF2 gene; and 12 months with mutations in the DKC1/NHP2 gene. The patients with mutations in the TERC/TERT/RTEL1/PARN gene had a significantly better transplant-free survival than those with mutations in the TINF2 or DKC1/NHP2 genes (p < 0.05 for paired comparisons; Fig. 5). Six patients underwent SLB. Patients who underwent SLB had significantly worse transplant-free survival than those without SLB (p = 0.042; Fig. 6a). Of these 22 patients, 14 received no medication, 6 received IS therapy (steroids and/or immunosuppressants), 1 received danazol, and 1 received acitretin. Worse survival was found in the patients who underwent IS therapy than those who did not (p = 0.012; Fig. 6b). There were no differences in radiological patterns, pulmonary function measurements, severity of BMF, or TMG variants between patients with IS therapy and without, or between patients with SLB and without except that the frequency of moderate to severe BMF in patients without IS therapy was significantly higher than those with IS therapy (p = 0.041) (Additional file 1: Table S2).

## Discussion and conclusions

DC is a rare genetic disorder of poor telomere maintenance characterized by a triad of dystrophied nails, skin pigmentation and mucosal leukoplakia. Pulmonary fibrosis is an uncommon complication of DC. To our knowledge, this is the first review to summarize the phenotype-genotype correlation and prognostic risk factors in patients with DC-related PF.

In this review, DC-associated PF occurred at a median age of 32 years old. About 40% of the patients presented respiratory symptoms after the age of 40. Moreover, 75% of these later-onset patients had normal complete blood counts or only mild thrombocytopenia, and one-third of them presented no mucocutaneous manifestations or only one triad feature. These findings suggest that it is not uncommon for PF to present without classic mucocutaneous features and BMF. Although DC is a genetic disease, half of DC-associated patients in this review had no family history. Therefore, high clinical suspicion and awareness of fibrotic interstitial lung disease due to DC are essential in all patients with unexplained PF, especially in those patients younger than 60 years old. Careful examination of mucocutaneous changes, hematological abnormalities, premature gray hair, or liver cirrhosis should be conducted to identify clues to telomere biology disorders. Further tests of peripheral blood telomere length and mutations in TMG are critical for differential diagnosis from other fibrotic interstitial lung diseases. Notably, the pulmonary function tests in DC-associated PF patients at diagnosis were on average severely compromised in this review. The reason why the diagnoses for these patients were delayed included failure to recognize this rare congenital disease especially in adult patients, and the late detection of PF in DC patients. Recently the study of baseline pulmonary function tests in patients with DC found that abnormalities were common even in the absence of overt pulmonary symptoms and associated with progression to significant pulmonary disease. Moreover, DC patients with clinically symptomatic PF had a rapidly progressive disease with a short survival [6]. These findings imply that pulmonary function tests and chest image are suggested to be performed at baseline and follow-up evaluation for all DC patients even without respiratory symptoms. Early recognition and management of DC-associated PF are crucial to delay the progression to the end-stage pulmonary disease.

The interstitial lung abnormalities seen on chest CT often show a UIP pattern in previously reported cases. However, half of the patients in this review showed an atypical pattern for UIP based on the presence of upper- or mid-lung predominance, multiple cysts, or diffuse ground grass opacity. No correlation between pathogenic gene mutations and radiological patterns was found. Why TMG mutations and resultant shortening of telomere result in radiological diversity needs to be further explored. Although various patterns were seen in chest CT in DC-related PF patients, histopathological UIP or probable UIP were found in two-thirds of patients, similar to the observation that, in 29 PF patients with TERT mutation carriers, the histopathological features were compatible with UIP in 86% and not classifiable in the rest [24]. These findings imply that TMG mutations maybe involved in the development of histological UIP.

We found that the common genes associated with PF in DC patients were TINF2, TERC, and DKC1. Mutations in PARN, which encodes poly (A)-specific ribonuclease—a deadenylase—have been linked with autosomal dominant familial PF and autosomal recessive Hoyeraal–Hreidarsson syndrome (a severe form of DC). Only biallelic, homozygous, or compound heterozygous variants of the PARN gene were reported in the Hoyeraal–Hreidarsson syndrome patients in literature [25–27]. To our knowledge, the heterozygous missense mutation located in exon 22 of PARN gene found in our case have never been reported in the DC-related PF patients. Recently Dodson et al. found that editing of a single PARN allele was sufficient to shorten telomeres, but that the same monoallelic PARN variants are not always associated with short telomeres; in other words, the impact of a given monoallelic PARN variant on telomere length in individuals is variable [28]. Therefore, the heterozygous variant in the PARN gene found in our case may contribute to the short telomere at the 30^th^ percentile of age-matched controls in this patient and needs further investigation.

Our review allowed us to examine how mutations in TMG were related to clinical phenotype and prognosis. The patients who had mutations in the TERC/TERT gene encoding the telomerase RNA and catalytic components developed BMF and PF much later than those with mutations in the DKC1/NHP2 gene encoding the box H/ACA ribonucleoproteins of telomerase and those with mutations in theTINF2 gene encoding the shelterin telomere protection complex. It has been reported that most patients with DC who have TINF2 gene mutations suffer severe consequences and have a higher morbidity of aplastic anemia before the age of 10 than those with other DC genes [29]. Our findings extend previous studies by demonstrating that DC patients with TINF2 gene mutations have an earlier onset and faster progression of PF than those with TERC/TERT gene mutations. In addition, we determined that DC patients with DKC1/NHP2 gene mutations also have an early onset of PF and have worse transplant-free survival than those with TERC/TERT/RTEL1/PARN gene mutations, while showing no differences from those with TINF2 gene mutations. These findings provide evidence for the role of gene mutations in predicting mortality from PF due to DC and may suggest that mutations in different components of telomere and telomerase may have different effects on the development and progression of PF.

The median survival time of DC-related PF patients was 2 years—the same as reported by Giri et al. in untransplanted patients with DC-related PF, shorter than the 2–3 years reported for IPF patients, and shorter than the 3.6 years reported for CHP patients with histopathological UIP [10,30]. Notably, the median transplant-free survival time of patients who underwent SLB was significantly shorter than those without SLB. Lung biopsy does not contribute to the diagnosis of DC-related PF. Therefore, we suggest that PF caused by DC should be excluded before diagnostic lung biopsy is undertaken in patients with unexplained PF.

There is currently no standard treatment for PF in DC. Several cases in our study were prescribed empiric IS therapy, but a worse survival was found in these patients than in those without IS therapy. Newton et al. found that IS therapy is associated with a higher composite endpoint of death, lung transplantation, hospitalization, or forced vital capacity decline for IPF patients with a telomere length below the 10th percentile (hazard ratio, 2.84; 95% confidence interval, 1.02–7.87) [31]. Our review confirms previous findings and further implies that IS therapy may be harmful to DC-related PF patients. It is suggested that first-line therapy for individuals with short telomeres and a UIP phenotype should include the anti-fibrotic agents, which have been shown to limit forced vital capacity decline and improve progression free survival [32]. However, Justet A, et al. found that a beneficial effect of pirfenidone on lung function decline could not be demonstrated in IPF patients with a TERC/TERT mutation [33]. The efficacy of antifibrotic medicine in DC-related PF patients need to be further evaluated in prospective studies. Androgen therapy might be a promising treatment on DC-related PF by ameliorating progressive telomere attrition in vivo. Marked improvement in the clinical and laboratory parameters of the pulmonary disease was demonstrated in a patient suffered from PF related to TINF2-associated DC after initiation of therapy with Danazol [13].

There are several limitations to this systematic review. First, we excluded one article that was in a language other than English or Chinese and one article without an available full-text version. Only case reports or case series of DC patients with PF in which detailed clinical data was reported were included. We may therefore have missed some relevant case reports. Second, our study design was a retrospective review of the cases reported in the literature, and a selection bias should therefore be acknowledged. Moreover, not all reports gave sufficient detail regarding the radiological findings, histopathological findings, and treatment follow-up data.

Third, telomere length was not measured in the majority of the patients in our study. Therefore, the relationship between the degree of telomere shortening and clinical phenotypes and prognosis cannot be explored. Fourth, the sample size of our study was small because DC-related PF is a rare fibrotic interstitial lung disease. Further prospective studies with larger sample sizes are therefore needed.

It is common for DC-associated PF to occur later in life in a “cryptic” form. PF caused by DC should therefore be kept in mind by clinicians in the differential diagnosis of patients with unexplained PF. Various radiological patterns were seen on chest CT in DC-related PF patients. Gene mutations in different components of telomere and telomerase are associated with the development and progression of PF. SLB and IS therapy may have harmful effects on survival and should be avoided in DC-related PF patients.

## Supplementary Information

**Additional file 1.** Clinical characteristics of patients with available follow-up data according to gene mutations, immunosuppression therapy, or surgical lung biopsy.

## Data Availability

The datasets generated and/or analyzed during the current study are available in the Mendeley repository, https://data.mendeley.com/datasets/7g23bwzzpz/1.

## Abbreviations

DC: Dyskeratosis congenita
TMG: Telomere maintenance genes
BMF: Bone marrow failure
PF: Pulmonary fibrosis
CT: Computed tomography
UIP: Usual interstitial pneumonia
IS: Immunosuppression
NSIP: Nonspecific interstitial pneumonia
SLB: Surgical lung biopsy
