# Supplementary material for: Pulmonary fibrosis in dyskeratosis congenita: a case report with a PRISMA-compliant systematic review
Source: BMC Pulm Med. 2021 Sep 3;21:279. doi: 10.1186/s12890-021-01645-w (PMC8418029; doi:10.1186/s12890-021-01645-w)
Supplement: Supplementary file 1 — Additional file 1. Clinical characteristics of patients with available follow-up data according to gene mutations, immunosuppression therapy, or surgical lung biopsy. [file 12890_2021_1645_MOESM1_ESM.pdf]

e-Table 1 Clinical characteristics of patients with available follow-up data according to gene mutations (n=16)

Variable	TERC/TERT/RTEL1/PARN (n=8)	TINF2 (n=5)	DKC/NHP2 (n=3)	P
BMF (moderate to severe）	3/5 (60.0)	3/5 (60.0)	1/3 (33.3)	1.000
CT patterns				
UIP /possible UIP	4/8 (50.0)	2/5 (40.0)	2/3 (66.6)	1.000
PFT				
FVC^{+}, %pred	40±3	36±18	41±4	0.905
Immunosuppression therapy	0/8 (0.0)	2/5 (40.0)	1/3 (33.3)	>0.05$^{\#}$
SLB	1/8 (12.5)	1/5 (20.0)	2/3 (66.6)	>0.05*

Data are presented as mean± standard deviation or number / total number of the patients with available data (%). BMF: bone marrow failure; CT: computed tomography; UIP: unusual interstitial pneumonia; PFT: pulmonary function test; FVC: forced vital capacity; SLB: surgical lung biopsy. $^{+}$ FVC was available in 2 patients with TERC/TERT/RTEL1/PARN variants, 3 patients with TINF2 variants and 2 patients with DKC/NHP2 variants. $^{\#}$ TINF2 vs TERC/TERT/RTEL1/PARN, p=0.128; TINF2 versus DKC/NHP2, p=1.000; TERC/TERT/RTEL1/PARN vs DKC/NHP2, p=0.273; * TINF2 vs TERC/TERT/RTEL1/PARN, p=1.000; TINF2 versus DKC/NHP2, p=0.464; TERC/TERT/RTEL1/PARN vs DKC/NHP2, p=0.152.

e-Table 2 Clinical characteristics of patients with available follow-up data according to immunosuppression therapy or surgical lung biopsy (n=22)

Variable	IS (n=6)	No IS (n=16)	P	SLB (n=6)	No SLB (n=16)	P
BMF (moderate to severe）	1/6 (16.7)	10/13 (76.9)	***0.041***	2/6 (33.3)	9/13 (69.2)	0.319
CT patterns						
UIP /possible UIP	2/5 (40.0)	9/16 (56.2)	0.635	2/3 (66.6)		
PFT						
FVC^{+}, %pred	43±10	39±12	0.655	45±8	36±12	0.249
DLCO $^{\#}$, %pred	24±7	39±14	0.106	31±7	38±17	0.434
TMG variants						
TINF2	2/3 (66.7)	3/13 (23.1)	0.214	1/4 (25.0)	4/12 (33.3)	1.000
DKC1/NHP2	1/3 (33.3)	2/13 (15.4)	0.489	2/4 (50.0)	1/12 (8.3)	0.136

Data are presented as mean± standard deviation or number / total number of the patients with available data (%). IS: immunosuppression therapy; SLB: surgical lung biopsy; BMF: bone marrow failure; CT: computed tomography; UIP: unusual interstitial pneumonia; PFT: FVC: forced vital capacity. $^{+}$ FVC was available in 3 patients with IS therapy, 6 patients without IS therapy, 4 patients with SLB and 5 patients without SLB. $^{\#}$ DLCO was available in 3 patients with IS therapy, 9 patients without IS therapy, 5 patients with SLB and 7 patients without SLB.
